# Supplementary material for: Specific dynamic variations in the peripheral blood lymphocyte subsets in COVID-19 and severe influenza A patients: a retrospective observational study
Source: BMC Infect Dis. 2020 Dec 1;20:910. doi: 10.1186/s12879-020-05637-9 (PMC7705851; doi:10.1186/s12879-020-05637-9)
Supplement: Supplementary file 1 — Additional file 1 : Supplementary Table 1. Dynamic variations in cell counts of total WBCs, total lymphocytes, and lymphocyte subsets in patients with non-severe COVID-19, severe COVID-19, and severe influenza A at weeks 1, 2, 3, and 4. Values represent P values. [file 12879_2020_5637_MOESM1_ESM.docx]

**Supplementary Table 1**. Dynamic variations in cell counts of total WBCs, total lymphocytes, and lymphocyte subsets in patients with non-severe COVID-19, severe COVID-19, and severe influenza A at weeks 1, 2, 3, and 4. Values represent P values.

| Patient  Groups/Immune cell population | | Wk1 -Wk2 | Wk1-Wk3 | Wk1-Wk4 | Wk2-Wk3 | Wk2-Wk4 | Wk3-Wk4 |
| --- | --- | --- | --- | --- | --- | --- | --- |
| Severe COVID-19 | WBC | 0.013 | 0.003 | 0.001 | 0.358 | 0.391 | 0.988 |
|  | LY | 0.646 | 0.013 | 0.002 | 0.001 | <0.001 | 0.719 |
|  | T cells | >0.999 | 0.214 | 0.034 | 0.161 | 0.003 | 0.167 |
|  | CD4^+^ T cells | 0.871 | 0.368 | 0.020 | 0.185 | 0.001 | 0.114 |
|  | CD8^+^ T cells | 0.703 | 0.461 | 0.330 | 0.185 | 0.060 | 0.606 |
|  | CD4^+^/CD8^+^ ratio | >0.999 | 0.683 | >0.999 | 0.414 | 0.440 | 0.636 |
| Non-severe COVID-19 | WBC | 0.412 | 0.005 | 0.210 | 0.044 | 0.557 | 0.230 |
|  | LY | 0.079 | 0.004 | 0.049 | 0.138 | 0.568 | 0.642 |
|  | T cells | 0.265 | 0.504 | 0.984 | 0.045 | 0.236 | 0.406 |
|  | CD4^+^ T cells | 0.457 | 0.607 | 0.536 | 0.133 | 0.136 | 0.838 |
|  | CD8^+^ T cells | 0.670 | 0.482 | 0.962 | 0.204 | 0.590 | 0.565 |
|  | CD4^+^/CD8^+^ ratio | 0.738 | 0.444 | 0.908 | 0.598 | 0.876 | 0.556 |
| Severe influenza A | WBC | 0.707 | - | - | - | - | - |
|  | LY | 0.102 | - | - | - | - | - |
|  | T cells | 0.001 |  |  |  |  |  |
|  | CD4^+^ T cells | 0.002 | - | - | - | - | - |
|  | CD8^+^ T cells | 0.007 | - | - | - | - | - |
|  | CD4^+^/CD8^+^ ratio | 0.550 | - | - | - | - | - |

Abbreviations: WBC: White blood cells, LY: Lymphocytes, *P <* 0.05 is statistically significant
